# Is Illicit Substance Use Gender-Specific? The Basic Points of Mental and Health Disorders

**DOI:** 10.3390/toxics10070344

**Published:** 2022-06-22

**Authors:** Ekaterina Georgieva, Krasimira Benkova, Nadya Vlaeva, Yanka Karamalakova, Radostina Miteva, Hristo Abrashev, Galina Nikolova

**Affiliations:** 1 Department of General and Clinical Pathology, Forensic Medicine, Deontology and Dermatovenerology, Faculty of Medicine, Trakia University, 11 Armeiska Str., 6000 Stara Zagora, Bulgaria; ekaterina.georgieva@trakia-uni.bg (E.G.); radostina.dimitrova@trakia-uni.bg (R.M.); 2 Department of Medical Psychology, Social Activities and Foreign Languages, Medical Faculty, Trakia University, 11 Armeiska Str., 6000 Stara Zagora, Bulgaria; krasimira.benkova@trakia-uni.bg (K.B.); nadya.vlaeva@trakia-uni.bg (N.V.); 3 Department of Chemistry and Biochemistry, Medical Faculty, Trakia University, 11 Armeiska Str., 6000 Stara Zagora, Bulgaria; yanka.karamalakova@trakia-uni.bg; 4 Department of Vascular Surgery, Medical Faculty, Trakia University, 11 Armeiska Str., 6000 Stara Zagora, Bulgaria; hristo.abrashev@trakia-uni.bg

**Keywords:** addiction, drug abuse, gender biology, neurotransmitter, vulnerable population

## Abstract

Among the groups of users of illicit substances, a high percentage are persons deprived of their liberty; at the same time, each social and age group is also affected, to one degree or another. The purpose of this study is to provide general data on the relationship between different psychostimulants, clinical and socio-demographic studies, and gender, both among the general population and in one of the most at-risk groups. This review identifies the use of illicit substances as gender-specific in the general population. A detailed study of the causal relationship between the use of illicit substances and gender was carried out. Electronic databases Academic Search Complete, PubMed, HealthCare, Web of Science, and Google Scholar were searched for relevant studies up to 2022 associated with drug abuse and mental and health disorders. The analysis indicated that the human population showed significant differences between the sex of the consumer as to the type of drug consumers, development of addiction, and relapse. We focus on the pathological changes caused by drug use, the personal and physiological individual traits that influence drug choice, and the extent of use in one of the most affected groups of individuals. The study may provide some guidance in developing gender-specific treatment and prevention, including response to some pharmacological and behavioral therapies. The review is intended for a wide audience of social workers, toxicologists, and pharmacologists.

## 1. Introduction

Drug addiction is characterized by dysregulation of emotional processes involved in motivation and stress patterns [1,2]. Social and cultural trends also affect and influence processes such as use, addiction, and relapse, as in men and women. There are significant differences between the sex of the users in terms of the type of drug user, the development of addiction, and relapse [3]. The stages of abuse include first use, followed by escalation, addiction with subsequent withdrawal to relapse, which is a common structure for both sexes and is the same for all drugs [4]. Each use begins with the acquisition of drugs that can lead to addiction in the vulnerable groups, which include the homeless, prostitutes, school leavers, children from families with abusers, and young people with antisocial behavior or depressive disorders. In addition, important factors are genetic predisposition, brain characteristics, psychological factors, exposure to physical, sexual, or emotional violence or trauma, substance use or family dependence, and use of alcohol, nicotine, or other drugs at an early age. These groups are represented by individuals with similar characteristics within a certain population, and as a result, drug addiction increases the risk of deteriorating health: physical, mental, and emotional [5]. Addiction can occur in certain individuals due to specific differences such as age, biological characteristics, and psychological factors, which can generate a pathological response to drugs [6]. This pathological response would make the desire to use drugs much stronger in some individuals, which increases the likelihood of developing drug addiction [7]. Drug use is associated with disorders of physical and mental health (depression, anxiety disorders) and, at the same time, a change in personal characteristics. The drug-related effects can be biologically mediated and result from the recreation of behavioral patterns or changes in social roles that affect the typical way of thinking, feeling, and acting. As an example, higher levels of neuroticism are associated with an increased risk of alcohol, marijuana, ecstasy, heroin, and tobacco use. Low self-esteem is associated with alcohol and marijuana abuse, and low life satisfaction is related to increased alcohol and heavy drug use [8]. 

Population and clinical studies describe the relationship between negative life events such as parental loss, divorce, sexual and physical violence, and victimization [9] and the increased risk of drug abuse due to early mental and physical trauma [10,11]. Stress ranks among the wide variety of causes and well-known risk factors leading to drug addiction and relapse, with addictive disorders being chronic and recurrent [12]. Childhood trauma is a risk factor for anxiety, mental disorders development, and substance use [13], which are more common in women [14,15]; therefore, drug addiction is more pronounced in them than in men [16]. The data show an increased use of illicit drugs such as opioids among women. In addition, there is a risk of opioid overdose and the rate of hospitalization, which means that drugs used, as well as heroin and other substances from the same chemical group, can be defined as “gender-specific” [17]. Women are more likely to increase the rate of heroin and methadone [18,19,20] and cocaine consumption than men [21]. Meanwhile, in the study of patterns of use and determining the average percentage of abuse: number of times abstinence and the number of times seizures during the period of addiction, no significant differences between the two sexes [22]. On the one hand, the psychological effects that drugs have on the human brain are closely correlated with the crimes committed. On the other, chronic and structural changes in the brains of addicts can lead to impulsive, antisocial, aggressive, and criminal behavior. There are two main directions: (1) committing a crime to finance drug use or due to psychopharmacology changes caused by already developed addiction; and (2) participation in crimes after drug use. Thus, consumers commit crimes, both to obtain funds for the purchase of drugs and under their influence. Smuggling, trafficking in illegal substances, theft, robbery, and prostitution are the main criminal activities carried out for financing and purchasing drugs [23]. Stimulants, such as cocaine or methamphetamine, have psychopharmacology effects that may increase the likelihood of crime and subsequent relapse [24]. Pierce et al. studied the phenomenon of “confusion” and concluded that drug use and committing a crime occur due to a common cause or causes with no direct causal link. Despite a highly controlled environment, illicit drug use continues in prisons, posing a high risk to consumer health [25,26]. Of the 17 EU member states, 9 countries (among which the Netherlands, United Kingdom, Latvia, Belgium, Finland, Hungary, Italy, Portugal, and Spine) report rates of over 50% in the use of illicit substances among detainees [27]. The prison society offers an incredible opportunity to study and analyze the problems related to drug use in general. For people who have used drugs intravenously, imprisonment is common. Prisoners are a risk group and are of particular interest in the transmission of blood-borne infections [28]. Human immunodeficiency virus (HIV/AIDS), hepatitis C (HCV), and sexually transmitted infections (STIs) are more common in prisons than in the general population [29,30,31,32].

Abuse and dependence on psychostimulants, mental illness, and infectious diseases are common problems among the prison population [33]. In most countries, the prevalence of HIV/AIDS in prisons is several times higher than in the general population, which shows a clear link between the incidence of HIV/AIDS among drug-addicted prisoners and the proportion of prisoners convicted of drug-related crimes [34]. Due to the nature of the group, the ability to control drug use is high, and implementing various addiction treatment programs would increase the success of the community. New approaches also need to be developed, taking into account possible neuroendocrine mechanisms that mediate differences in the physical effects of substance use in both sexes [35]. The main problem is that almost all drug treatment programs are designed for men. Thus, clinical strategies for working with drug addicts are focused mainly on men, while for women; this is the standard corrective practice. Women have special needs, which is why programs designed for men may be inapplicable and ineffective in their treatment [36,37]. 

This study aimed to provide general data on the relationship between drug use and demographic, economic, social, and physiological factors in vulnerable populations. Exploring this relationship is significant for understanding the complex mechanisms leading to the progression and disorder of drug abuse.

## 2. Materials and Methods

Our study aimed to provide general data on the relationship between different psychoactive substances. The present systematic review adopted within the study was based on screening and extracting the literature data on gender-based illicit substance use. The points of literature research included psychosocial, physiological, and medical approaches. The review protocol is in line with the system of systematic reviews and protocols for meta-analyses (PRISMA-P). A topic and a research question were identified and included: identification of a research question and relevant studies; development of a comprehensive search strategy; and database selection and results summarization [38]. The detailed studies were performed in the field of illicit substances, drug addiction, vulnerable groups, and basic factors of drug choice.

**Figure 1:**
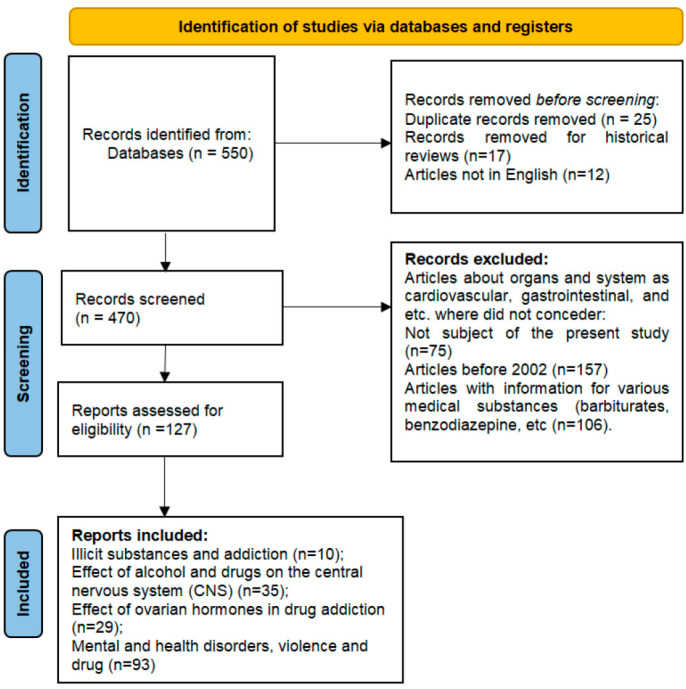
Comprehensive literature search strategy according to the chosen topic: inclusion and exclusion criteria.

A comprehensive literature search strategy was developed according to the chosen topic and according to PRISMA guidelines [38]. We identified more than 500 bibliographic reviews, clinical studies, literature, and systematic reviews in the electronic databases Academic Search Complete, PubMed, HealthCare, Web of Science, and Google Scholar for the bibliographical search and relevant scientific articles containing in its titles the keywords: “illicit substances”, “drug substance”, “addicted human brain and addiction”, “drug abuse”, alcohol abuse”, “stimulants”, “cocaine”, “amphetamine-type drugs”, “neurotransmitters”, “transmit signals from nerve cells”, “gender (or) man and woman at risk”, “dopamine receptors”, “gender biology”, “childhood trauma”, “post-traumatic stress”, and “mental health disorders”. The historical reviews, case reports, and articles that were not related to the objectives were rejected. We included 116 studies associated with drug abuse and mental and health disorders of the English language in the period from 2002 to 2022 inclusive. Presented in this systematic review are the effects that illicit substances have on the central nervous system, dopamine receptors, mental disorders, and transmission of infectious diseases in the risk group (see Figure 1).

### 2.1. Inclusion Criteria

The present review includes the scientific literature articles and reviews in English focused on: (i) general population and vulnerable group, their mental and health disorders; (ii) the link between the use of illegal substances without age and regional restrictions (see Figure 1 for inclusion criteria). 

In particular, we included studies focused on health risk factors and mental disorders; drug use in adults; dysregulation of emotional processes, including depression and post-traumatic stress disorder; children’s injuries; and how the choice of drugs depends on gender. An in-depth analysis of the English scientific literature in the period from 2002 to 2022 was conducted, which contained in its titles the keywords: “illicit substances”, “drug substance”, “addicted human brain and addiction”, “drug abuse”, “alcohol abuse”, “stimulants”, “cocaine”, “amphetamine-type drugs”, “neurotransmitters”, “transmit signals from nerve cells”, “gender (or) man and woman at risk”, “dopamine receptors”, “gender biology”, “childhood trauma”, “post-traumatic stress”, and “mental health disorders” and managed to include studies according to PRISMA guidelines [38].

### 2.2. Exclusion Criteria

Non-English articles were not included, as previous studies have shown no statistically significant change in the data. Studies and literature reviews describing the effect of various drugs on organs and systems such as cardiovascular, gastrointestinal, etc., were not considered; because they are not the subject of the present study.

## 3. The Early Childhood Trauma as the Start Points of Mental Disorders and Drugs Use

### 3.1. Mental and Health Disorders, Violence, and Drugs

Men and women are differently affected by specific types of childhood trauma. Studies on interpersonal trauma and drug abuse in both sexes show a strong link between the two conditions; women are at greater risk of use after suffering from interpersonal trauma [39,40]. Gender differences, childhood abuse, subsequent drug use, and crime were reported in both sexes. Significantly more childhood and adult abuse was reported in the group of women than in the group of men [16]. Therefore, the severity of drug abuse and its consequences can be a leading indicator of the rate of criminal activity among women. Hyman et al. [41] also investigated the gender-specific effects of childhood trauma by examining cocaine dependence and relapse in men and women. Authors found a correlation between emotional, sexual violence, and overall psycho-traumatic disability in childhood with an increased risk of relapse in female users compared to men. These findings suggest that childhood trauma increases the likelihood of cocaine recurrence and escalation of drug use after initial use in women but not men [41]. Cocaine activates central and peripheral stress pathways, with extrahypothalamic and hypothalamic corticotrophin-releasing factor (CRF) release systems involved in increasing cocaine dependence and frequent relapses [12,41]. Moreover, cocaine addicts were found to have higher levels of daily measured morning sex hormones progesterone and plasma cortisol during the first month of abstinence than healthy controls. Autonomic and noradrenergic abnormalities are also well documented with the overactivity of these systems during acute and prolonged cocaine withdrawal. The elevated levels of circulating glucocorticoids are associated with higher levels of self-administration of psychostimulants, including amphetamine and methamphetamine [12,42].

Mental disorders, loss of parental rights or the death of a partner or child, job loss, and divorce can be a prerequisite for abuse. Women are more likely than men to use multiple substances and alcohol, nicotine, medications, legal and illegal drugs, etc., to relieve physical or emotional pain or to deal with depression. Increased depressive symptoms in destructive mood dysregulation disorder, persistent depressive disorder, and premenstrual dysphoric disorder are associated with methamphetamine use. Similar to depression and panic disorders, women lead to use opiates compared to men counterparts [43]. 

Due to biological differences in metabolism and hormone levels (cortisol) between men and women, these actors are used as key points to assess risk behavior and differences between patterns of alcohol consumption in men and women [43]. Gender variation in alcohol use is very much influenced by different factors such as sociocultural (social habits and customs), sex-related, physiological, etc. Often, men drink in the context of pleasant emotions [44] but drink more and present more problems associated with alcohol use [43]. Women primarily reported using alcohol when experiencing negative moods and emotions [44]. Women with risks-problematic and harmful use and alcohol dependence can face more discrimination and health problems and sexual abuse, divorce, and unemployment. Binge drinking can increase the risk of serious social, emotional, and physical disorders. The early childhood trauma and early stress often precede the development of alcohol abuse and, related to this, greater negative consequences such as divorce, partner and child abuse perpetration, psychiatric hospitalization, incarceration, and homicide and suicide death. Cross and colleagues investigated the relationship between childhood trauma, PTSD, and alcohol addiction in largely low-income, African-American men and women with high self-reported childhood trauma. The results show that traumatic stress in early childhood was to both alcohol and substance use problems for both sexes, but problematic alcohol use effects were greater for men than for women [45]. 

Panic disorder is twice as common in women as in men and involves a risk of alcohol and drug addiction. Alcohol withdrawal and abstinence can cause panic attacks, with panic disorder may use alcohol to decrease panic symptoms. In addition, the link between panic disorder and smoking addiction has been determined. Daily nicotine use is associated with an increased risk of panic disorder in active smokers than in previous smokers of both sexes [46]. The use of alcohol and/or illicit drugs places women at increased risk of violent death in the home (murder of a spouse or partner) and suicide [47]. In addition, women are more likely to use a combination of alcohol and medications than men, especially narcotic analgesics and tranquilizers [48]. Gender has been found to affect cocaine use and panic attacks, increasing their incidence among men but not women. There are significant differences in the severity of panic attack symptoms in users of different drugs [46,49]. The main risk to consumer health is not limited to single-use. Repeated exposure may lead to addiction, which is characterized by forced use and loss of control over drug-related behaviors. 

Chronic drug use causes neuroadaptations in the brain and long-term disturbances in its structure and function [50]. Neurobiological changes with prolonged illicit drug use continue after discontinuation of the drug, which explains the high risk of recurrence in addicts of both sexes [51]. A more detailed understanding of the relationship between the type of drug and sex is crucial in the introduction of new institutional and medical-social programs to prevent the use of drugs or the treatment of drug addiction [52,53].

### 3.2. Drugs Use and the Transmission of Infectious Diseases

Illicit drug use, infectious diseases, and imprisonment are closely linked. Many studies report that between 56% and 90% of people who inject drugs are serving sentences at some point in their lives [54]. Furthermore, the act of imprisonment may also put the otherwise non-infectious at-risk group, for example, the prisoners. High-risk sexual behavior (including sexual violence), the use of common needles in intravenous drug administration, re-imprisonment, and tattooing in prisons [54,55], and lack of sterilization or reuse of medical or dental instruments [56] are factors responsible for the high incidence. HIV transmission is often accompanied by complications, including co-infection with tuberculosis (TB) and multidrug-resistant (MDR) tuberculosis (TB), as institutional foci of MDR-TB mainly affect HIV/AIDS-infected individuals [57,58,59]. Prisoners with HIV/AIDS and/or HBV infection are more likely to be infected with HCV due to the similarity in the routes of transmission of these infections. Zampino et al. reported in a study conducted among Italian prisoners that the prevalence of anti-HCV reached 89.6% in those infected with HIV/AIDS, while those without HIV/AIDS infection infected with HCV were 15.5% [60]. Another study reported a higher incidence of anti-HCV positivity in anti-HIV-positive patients than in anti-HIV-negative patients (65.5% vs. 27.5%). In addition, these data suggest that the probability of HCV infection among prisoners may be many times higher in places of detention than in the general population [61]. Moreover, the women are at higher risk of contracting HIV through unprotected sexual contact and intravenous drug administration. The number of HIV-positive women injecting drug users continues to increase in Asia and Eastern Europe [37]. They are increasingly being diagnosed as HCV-positive, which has recently correlated with more crimes committed by women and their imprisonment for drug-related crimes in general [60]. The most common HCV infections are in women aged ≥30 years and intravenous drug users. HCV-HIV co-infection is 1.2% in men and 1.5% in women, with the highest percentage among older prisoners and drug users [61]. High values among female prisoners have also been reported in Canada, Mexico, Honduras, Nicaragua, and Panama [62,63,64,65]. Monitoring the spread of HIV in prisons shows that the infection is a serious problem and requires immediate action.

## 4. Illicit Substances Use and Their Effects on Dopamine Receptors and Brain

### 4.1. What does Happen in Addicted Human Brain?

Drug and alcohol addiction is classified as a chronic relapsing condition [41,66]. Scientific approaches to addiction are usually based on the premise that addiction is a process that results from changes in the brain due to chronic drug use, which is primarily a social rather than a health problem [67]. The brains of women and men develop in response to genetic and hormonal signals, physical and emotional environment, and individual sociocultural experiences. These components are specified for the individual, but they all participate in brain development throughout life. As a result of biological processes and the complex social environment in which the individual is placed, there are differences in drug addiction by gender, as the brains of men and women differ in one way or another [68]. These differences are due to the response to genetic and hormonal signals, the physical and emotional environment, and individual sociocultural experiences [69]. Four types of gender differences are described: qualitative, quantitative, convergent, and gender differences in the population, which lead to variations in a given trait between men and women. These four types of gender differences operate within each person, and each type of gender difference contributes to the overall phenotype of the individual; the individual types of gender differences can shape the individual phenotype.

### 4.2. Drug and Alcohol Use, Addiction and Changes in Brain

Drug use is known to cause significant and lasting changes in brain chemistry and function [16]. The transition from drug experiments to addiction is accompanied by progressive changes in the brain called neuroadaptation [70]. The stages during which addiction develops are three and include intoxication, withdrawal, and preoccupation [70,71]. Neuroadaptation compromises brain function and can lead to a transition from controlled, accidental substance use to chronic substance use. These structural and functional changes of the central nervous system (CNS) promote and maintain drug addiction and contribute to relapse. Addiction might describe as a recurring cycle with three stages: binge/intoxication, withdrawal/negative effects, and preoccupation/anticipation. Each stage is particularly related to one of the areas of the brain described below [72]. 

The progression of addiction includes changes in normal brain circuits and long-term pathological and neuroplastic changes that involve critical neurotransmitters such as GABA, glutamate, dopamine, opioid peptides, serotonin, acetylcholine, and neurochains [73]. An example, by potentiating the GABAergic receptors, alcohol inhibits the function of this neurotransmitter and induces continuous stimulation of dopamine. It leads to the production of dopamine in the NAc, which increases the activity of the dopaminergic neurons and leads to desensitization of the reward systems [74].

### 4.3. Dopamine Neurotransmission

**Figure 2:**
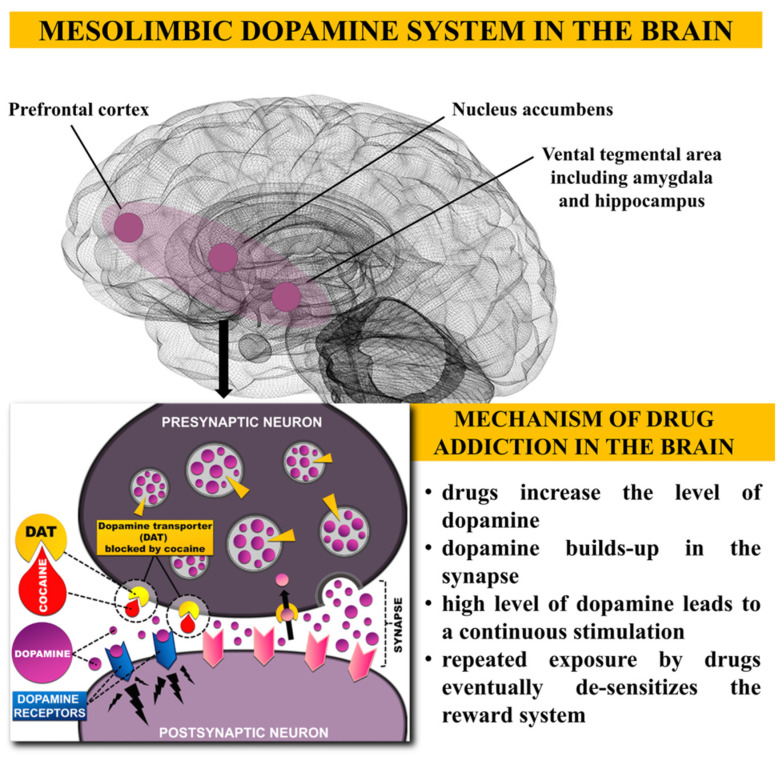
Model of drug addiction mechanism and dopamine projection in mesolimbic dopamine system: dopamine, drugs (stimulants, opioids), dopamine receptors, dopamine transporter (DAT). A schematic diagram illustrates the mesolimbic dopamine system in the brain and the dopamine neuronal role in drug addiction. Dopamine projections target the nucleus accumbens, prefrontal cortex, amygdala, and hippocampus. Direct administration of drugs and their systemic administration results in the release of large amounts of dopamine from dopamine neurons in the VTA, which is released into the nucleus accumbens and prefrontal cortex [77]. Reprinted with permission from Ref. [77]. 2021, NIDA Public Inquiries Team.

All addictive drugs activate the mesolimbic dopaminergic system through specific neurobiological schemes (Figure 2). The mechanism included large quantities of dopamine (D) from the dopamine neurons of the ventral tegmental area (VTA). These actions lead to the activation of dopamine neurons by blocking the dopamine transporter effect [75]. In addition, drugs increase the strength of conditioned reactions and the reactivity of stress [71]. Synaptic plasticity is the best-studied neuroadaptation that occurs after exposure to psychostimulants. This occurs at the synapse between two neurons and involves changes in receptor expression, signal transduction, or synapse structure. For example, the use of stimulants, such as cocaine, leads to synaptic rearrangement and possibly altered excitability of dendritic cells due to changes in their morphology [76]. The neurotransmitter dopamine (DA) plays a significant role in the increased sensitization of the stimulatory motivational properties of drugs [77]. Therefore, it is part of the most widely studied neurotransmitter system—dopaminergic. It is a well-established fact that drug use leads to increased dopaminergic transmission in the centers of the brain [78]. During drug intoxication or thirst, the frontal brain areas are activated. It is part of a complex model that includes brain circuits in the nucleus accumbens (NAc) areas, prefrontal cortex [79], amygdala and hippocampus, prefrontal cortex, and cingulate gyrus related to reward, motivation, memory, and cognitive control [80]. Thus, when used, there is an increased mediated response in the striatum and amygdala and weakened activity in the prefrontal cortex. Decreased inhibitory control of the prefrontal cortex to the hyperactive amygdala-striatum system has been observed. The individual cannot self-regulate drug-seeking behavior, which leads to constant and forced use, regardless of the negative consequences [76,81].

#### D1- and D2-Like Family of Dopamine Receptors

Brain imaging studies show that addiction is associated with abnormal functioning of the ventromedial cortex, amygdala, striatum, anterior brain, and insular/somatosensory cortex, as well as nonspecific neurotransmitter systems that modulate the activities of neuronal activities involved in decision-making processes [76,82,83]. The results for opiates, ethanol, nicotine, amphetamine, and cocaine show increased concentrations of extracellular dopamine in both zones, but mostly in NAc. PET and fMRI imaging show that cue exposure to cocaine and nicotine administration induces activation of the amygdala region. The reinforcing effect of the drug remains formatted salient stimuli and internally rewarding events, which are due to long-lasting cellular and molecular adaptations. The stimulation of dopaminergic neurons and increase in glutamate release facilitated the alterations in intracellular processes by increasing or decreasing the synthesis of messenger, transcription, and or structural proteins and mediation of drug-induced sensitization [84,85,86]. Alcohol interacts with the dopaminergic, serotonergic, glutamatergic, and GABAergic neurotransmitter systems in the CNS. In addition, it is responsible for brain modulation and is also present in the reward system. These interactions result in reward, stress effects of circuits reinforcing, and cause changes in neuronal function that underlie the development of alcoholism [74]. Alcohol consumption produces increased levels of DA outside neurons in the ventral tegmental area, and its use discontinuation produces a decreased level of the neurotransmitter, which may contribute to symptoms of alcohol relapse and withdrawal in dependent individuals [87]. For example, the human brain investigation by positron emission tomography (PET) has shown that drug and ethanol intoxication leads to the release of DA and opioid peptides into the ventral striatum area [73,74].

Under normal conditions, the brain maintains a delicate balance between the effects controlled by the dopamine receptors from D1 and D2-like family. Their drug-related activation can lead to stimulation or inhibition of various signaling pathways [88,89,90]. Stimulants cause a rapid increase in extracellular DA levels and supraphysiological activation of the dopamine receptor [91]. Effects caused by cocaine are due not only to DA increase concentration but also to the subsequent stimulation of dopamine D1R and particular D2R receptors. A fast and steep increase in DA levels is associated with activating low-affinity D1R, which are associated with drug rewarding effects, while stimulation of high-affinity D2 receptors is not sufficient for the drug reward effect [73]. The main biochemical mechanism of addiction is due to poor D2R binding and dopamine release in the striatum regardless of the substance [90]. The D1R and D2R are expressed in medium spiny neurons (MSNs) in the striatum, with opposite intracellular effects on cAMP signal transduction [92]. In turn, D1Rs activate the enzymatic activity of adenylyl cyclase and thus alter gene expression, membrane stabilization, and synaptic plasticity [93], and the D2R pathway plays a major role in inducing relapse in cocaine and dominates in cocaine-related signals or stress [94].

### 4.4. Neuroplasticity Changes and Other Pathways in Drug and Alcohol Addiction

Serotonin is another neurotransmitter that is involved in the stages of drug abuse and addiction, including cocaine, amphetamines, LSD, and alcohol. Changes in serotonin levels and the serotonin pathway dysregulation are implicated in the pathophysiology of mood and anxiety disorders and can cause not only obsessive-compulsive disorder, anxiety disorders, and depression but drug and alcohol addiction and relapse. The psychostimulants have significant effects on non-dopaminergic mechanisms and monoamine levels such as 5-hydroxytryptamine (5-HT), indicating a role for 5-HT in drug reward. Stimulant administration inhibits monoamine reuptake of serotonergic neurons, elevating extracellular 5-HT in a dose-dependent manner in brain regions NAc, ventral tegmental area, dorsal raphe nucleus, hippocampus, striatum, and cortex. It is known that the acute self-administration of cocaine and other stimulants such as amphetamine and methamphetamine produce acute stimulatory effects on forebrain 5-HT levels. The elevation of the extracellular level of serotonin 5-HT is accompanied by increased activity of the 5-HT synthesizing enzyme tryptophan hydroxylase in the raphe nucleus and subsequent autoreceptor-mediated inhibition of raphe firing [95].

The gamma-aminobutyric acid system is the third neurotransmitter pathway that is especially important for understanding drug and alcohol addiction. Glutamate is a major excitatory neurotransmitter with a high concentration in brain tissue and plays an important role in amphetamine and its derivatives addiction (ATS). Acute and chronic application of ATS leads to over-activation of the NAc dopamine neurons and can change neural plasticity by change of the functions of multiple members GABA produces in the mesolimbic circuit. The interacting process includes GABAergic interneurons and GABAergic projection neurons and glutamate activation, increased dopamine release through the mesolimbic and mesocortical pathways in the VTA, prefrontal cortex, and striatum to the NAc, which can lead to sustained adaptive and pathological changes in regions with GABAergic neurons and stable drug addictive state [96]. The actions of glutamate are mediated by two kinds of receptors: fast-acting ligand-gated ion channels, which include N-methyl-D-aspartate (NMDA) etc., and slow-acting G-protein (mGlu) receptors. The compensatory glutamate-receptor responses might trigger the DA-receptor adaptations with the potential to affect synaptic plasticity. Activation of these receptors leads to stimulation of intracellular signaling pathways, inducing glutamatergic neurotransmission and drug-induced plasticity. An example of glutamate transmission during drug use (cocaine, nicotine, alcohol, and heroin) is blocking DAT from cocaine, which leads to increased DA levels and activates presynaptic or postsynaptic D1 dopamine receptors. The high concentration of DA can activate postsynaptic D1 receptors and increase NMDA-mediated glutamate signaling by cross-talk between D1 and NMDA receptors [97].

Ethanol increases the GABA by acting on the signal-receiving neuron and facilitates the activity of the GABAA receptor, causing an increase in GABA release in the nucleus accumbens and amygdala. The alcohol acute reinforcing action is carried out by inhibition of glutamate activity in the brain, causing a drop in the extracellular glutamate levels in a striatum. Parallel with this, chronic alcohol exposition can lead to the expression of genes that encode components of the GABAA receptor, which changes the GABAA receptor’s function. The increase in the levels of neurosteroids involves allosteric modulation of GABA and changes the excitability of neurons. It is known that glutamate exerts its effects through the NMDA, and high glutamate activity leads to excessive alcohol consumption. The transmission of glutamate is most likely affected due to alterations in the NMDA receptor’s functions, and this is involved in changing neuroplasticity [74].

Drug intoxication may be a start point of the neuroplasticity changes that can trigger longer-term molecular neuroadaptations via transcription factors and modify gene expression. Epigenetic remodeling in the brain during drug use involves excessive dopamine signaling, which modulates gene expression and alters synaptic function and chain activity [98]. In case of long drug use, gene expression can induce changes in the brain and transition to addiction in the vulnerable. Over time, this can be related to maladaptive behaviors in drug abuse individuals. An increasing number of studies have investigated epigenetic alterations, which are a result of drug-induced gene expression changes such as histone modifications, DNA, and miRNAs methylation. For example, administration of morphine can cause histone acetylation and methylation, and DNA methylation, which lead to changes in gene expression. In the phase of cocaine withdrawal, a large number of gene promoters are hyper-methylated, and after self-administration of the drug, they become hypo-methylated during the reinstatement use stage [99]. In case of chronic exposure to various drugs, activated cAMP/PKA signaling pathway and the up-regulation of a postsynaptic Gs/cAMP/PKA in NAc. An example of critical neuroadaptation is dopamine-mediated cocaine addiction, which promotes escalations of cAMP-dependent protein kinase A (PKA) activity and PKA-dependent protein phosphorylation that modulate the cAMP formation [73]. Fos family proteins (c-Fos, FosB, Fra1, and Fra2) are induced rapidly and transiently in specific brain regions such as the nucleus accumbens and dorsal striatum in acute administration of many drugs of abuse. Chronic drug administration leads to an abnormally and excessively high level of the ΔFosB expression, which characterizes the transcription factor ΔFosB as a sustained molecular trigger for initiating and maintaining an addiction state. It could sustain changes in gene expression and causes increased sensitivity to the behavioral effects of drugs of abuse that persist long after the last drug administration [100]. The repeated exposure to cocaine, amphetamine, morphine, nicotine, phencyclidine, and alcohol leads to the accumulation of stable biochemically modified isoforms of ΔFosB within the same brain regions. By dimerization of 35 to 37 kDa isoforms of ΔFosB with JunD has formed an active and long-lasting AP-1 complex and provides drug-induced changes in gene expression for long periods of drug withdrawal in NAc [98].

## 5. Does the Choice of Drugs Depend on Gender Biology?

According to the World Drug Report 2019, the use of drugs from the group of stimulants (cocaine type, cocaine salt, ATS) has increased in Western and Central Europe compared to the Southeastern and Eastern regions (Table 1). In the same year, approximately 20 million of the total population (between 17 and 25 million) or 0.4% of the adult population between the ages of 15 and 64 years used cocaine 27 million people took at least one synthetic stimulant of amphetamine-type (ATS) [101]. ATS is the group of synthetic substances, including amphetamine, methamphetamine, ecstasy, and their derivatives, which are taken mainly in tablet form but can also be inhaled, smoked, or injected [102].

Women are usually more likely to provide specific reasons for drug use in general and are more likely to be diagnosed with major depression [102]. For example, past trauma has been significantly associated with cocaine recurrence in women but not men [14]. In addition, women increase the rate of opioid and cocaine consumption faster and develop addiction faster than men. Men are more likely to work, hold higher positions, support themselves and use stimulants as part of a larger model of antisocial behavior [101,103]. However, no statistically significant differences between men and women were observed in the study of use patterns and the determination of the average use rate, the number of abstinence times, and the number of seizures during the period of addiction [104]. 

In addition to differences in socio-demographic characteristics, the prevention and successful treatment of drug addiction must pay particular attention to gender-based biological differences. More and more women are abusing illicit substances, which is why studies focus on important differences in the biology of the sexes and substances they use, the epidemiology of substance use disorders, etiological considerations, and psychiatric comorbidity [21]. It has been established that the drug used by both sexes are not the same [105,106]. The two sexes show different substance preferences from varied chemical groups and specific pharmacological characteristics [107]. Drug abuse and prescription misuse of drugs are generally more prevalent in males than females (Table 2 and Table 3). According to National Center for Drug Abuse Statistics, 22% of males have used drugs, while for women, this percentage was 17% in 2021 [108].

In developed countries, drug use increased far rapidly from 2000 to 2018, among which cannabis was the most used substance worldwide in 2018 [108].

In the study of the relationship between sexual disorders and drug use (most common opioids, cannabis, and alcohol), the telescopic progression has been observed [109,110]. This phenomenon is characterized by accelerated progression and includes the onset of substance use, dependence, and first treatment. Although women use less of the substance and have used it for a shorter period of time, they have a more severe clinical picture than men [46]. Following intranasal cocaine use, they have higher ‘nervous’ reactions, become more addicted, meet the criteria for substance use more quickly and enter treatment programs earlier than men [111]. One of the main emphases in research related to the use of various drugs is the influence of sex hormones. They can increase the positive effects such as greater euphoria, increased energy, and intellectual efficiency of stimulants use during the early stages of addiction [112]. 

Preclinical studies have shown that the levels of the two hormones progesterone and estrogen affect the enhancing effects of stimulants [113,114]. The positive enhancing effects of cocaine and amphetamines are more intense during the follicular phase when estradiol is elevated than in the luteal phase of the menstrual cycle when both estradiol and progesterone increase. This is probably due to the opposite and direct effects of estrogen and progesterone on the neurotransmitters serotonin, dopamine, and norepinephrine, which suggests that stimulants may cause various effects in different phases of the women’s menstrual cycle [115,116]. During the luteal phase, progesterone production attenuates these effects, and the presence of estrogen in the follicular phase increases them [117]. Fox et al. found that cocaine-dependent women showed significantly higher levels of salivary progesterone and lower estrogen/progesterone ratios during the menstrual cycle [112]. Thus, the menstrual cycle can contribute to a faster escalation of drug use. The elevated levels of hormonal markers are indicative of hormonal adaptations in response to cocaine withdrawal. The elevated luteal progesterone in addicts is associated with lower stress and thirst caused by drugs [117]. In men, testicular hormones and estradiol do not affect cocaine intake, which is characterized as a gender difference and is due to sexual differentiation in early brain development [118].

## 6. Discussion

The use of drugs, craving, and relapse has important implications for public health and social policy and cause significant harm to society [119]. Drug addiction is characterized as a multifactorial phenomenon, which is manifested by dysregulation of the emotional processes of reward patterns involved in motivation and stress patterns [120]. 

The aim of this study is to describe psychological and physiological factors related to drug craving and relapse in humans and the role of sex hormones in drug reward and addiction. A majority of the clinical studies reviewed the drug craving and relapses as unspecifiable for sexes and do not support the notion of sex differences and ovarian hormones’ role during early abstinence, where women may be more vulnerable. Nevertheless, recent studies are increasingly emphasizing the relationship between drug use and sex differences [121,122,123]. Nicolas et al. review clinical studies descriptive the role of sex differences in drug (psychostimulants and opioids) craving and relapse and consider drug-seeking after the extinction of drug self-administration and incubation of drug craving in initiation and escalation of drug self-administration and withdrawal. They also discuss the role of ovarian hormones in cocaine craving and relapse/reinstatement and summarize study results on the human brain’s response to drug cues and stress in both sexes [119]. Stress ranks among a wide variety of causes, and it is a well-known risk factor leading to drug addiction and relapse [45]. Men and women are differently affected by specific types of trauma and traumatic life experiences. There is much evidence to show a close link between mental disorders, which can lead to drug abuse. On one side, depression, post-traumatic stress disorder, and severe mental illness can be the basis for initiating drug abuse; on another side, abstinence from alcohol, sedatives, stimulants, and opiates increases the risk for depression and anxiety [11]. An important hormonal feature leading to a reduction in cocaine-induced craving is the level of progesterone, while estradiol has the opposite effect. Some studies have reported that high levels of endogenous progesterone lead to a reduction in the adverse effects of mental stress in cocaine-induced desire to use, while other studies do not confirm this dependence [119]. For example, women with high hormone levels are less sensitive to cocaine-induced craving than those with low progesterone levels. Yonkers et al. show that the progesterone treatment would reduce cocaine use or relapse during the postpartum period in women. The reduction in drug use during pregnancy and recurrence in the postpartum period coincides with an increase/decrease in progesterone levels [124]. According to the literature, there is still no clear evidence that ovarian hormones play a critical role in that women are more vulnerable to psychostimulant as cocaine and methamphetamine [125] and opioid (heroin) craving and relapse [126,127]. In addition to hormone levels and menstrual phases [119], the use and development of drug addiction is influenced by complex social, cultural, and economic factors, which further contribute to the degree and prevalence of self-reported craving and relapse rates [4].

## 7. Conclusions

Detailed knowledge of the psychological profile of sexes, biological features, and hormonal differences would contribute to the development of complex behavioral and drug therapy for drug and alcohol addiction, craving, and relapse in vulnerable groups. Treatment may be more successful, especially when it is voluntary and controllable. Strengthening the internal motivation of consumers should include determining the individual psychological and social status of each consumer personally, which requires the exclusion of programs aimed at the general population. The present review can serve as a basis for the development of gender-specific treatment, consistent with the biological mechanisms underlying gender differences.

## Figures and Tables

**Table 1:** Data trend of illicit substances use in general population for according to World Drug Report 2019: large decrease in use drug type is reported for Greece, Latvia, and Lithuania for 2019 (*); large decrease in use drug type is reported for Ukraine for 2019 (**) [101].

Regions	Drug Type	Trend
Western and Central Europe	Cannabis	Large increase/large decrease *
	Cocaine	Large increase
	ATS	Large increase/large decrease *
	Opioids	Large increase
	Heroin	Large increase
	Hallucinogens	Large increase/large decrease *
	LSD	Large increase
Southeastern Europe	Cannabis	No data
	Cocaine	No data
	ATS	No data
	Opioids	No great change
	Heroin	No data
	Hallucinogens	No great change
	LSD	No great change
Eastern Europe	Cannabis	No data
	Cocaine	No great change
	ATS	No great change
	Opioids	No great change
	Heroin	No great change
	Hallucinogens	Large decrease **
	LSD	Large decrease **

**Table 2:** World data on drug abuse by sex or gender according to National Center for Drug Abuse Statistics in population aged 15–64 [108].

Drug Abuse	Man, %	Woman, %
Opioids	4	3.5
Heroin	0.5	0.2
Cocaine	2.6	1.5
Cannabis	18.5	13.5
Methamphetamines	0.8	0.4
Misuse prescription pain killer pills	3.9	3.4
Misuse prescription tranquilants	2.2	2.0
Misuse prescription sedatives	0.5	0.5

**Table 3:** The data on drug abuse by sex or gender during 2018, according to Statistical Annex of United Nations Office on Drugs and Crime in population aged 15–64 in some European countries [108].

	Man, %	Woman, %
COUNTRY	Cannabis	
Estonia	9.20	4.81
Finland	11.20	5.20
Germany	8.87	5.25
Netherlands	13.90	6.30
Norway	7.00	3.70
United Kingdom (England and Wales)	10.28	5.00
	Cocaine	
Estonia	1.32	0.83
Finland	1.40	0.50
Germany	1.41	0.82
Netherlands	2.70	1.30
Norway	2.10	0.20
United Kingdom (England and Wales)	4.10	1.76
	Amphetamine and Methamphetamine	
Estonia	1.65	0.45
Finland	2.50	0.90
Germany	1.50	0.90
Netherlands	1.80	0.90
Norway	1.10	0.10
United Kingdom (England and Wales)	0.76	0.39
	Illicit opioids ^●^/Prescription opioids ^●●^	
Estonia	0.33^● ^/0.22 ^●●^	0.08^● ^/0.08 ^●●^
Finland	0.90 ^●●^	0.80 ^●●^
Germany	0.48 ^●^	0.39 ^●●^
Netherlands	No data	No data
Norway	No data	No data
United Kingdom (England and Wales)	No data/0.07 ^●●^	No data/0.04 ^●●^
	Barbiturates and Benzodiazepines	
Estonia	0.88	0.23
Finland	No data	No data
Germany	No data	No data
Netherlands	7.00	13.30
Norway	No data	No data
United Kingdom (England and Wales)	0.57	0.23

## Data Availability

Not applicable.
